# Influencing factors of hospital-acquired COVID-19 prevention and control status among emergency support frontline healthcare workers under closed-loop management: a cross-sectional study

**DOI:** 10.3389/fpubh.2023.1209646

**Published:** 2023-07-27

**Authors:** Man Luo, Guoqin Xia, Tieer Gan, Zhifang Zhao, Jiannong Wu, Ting Hu, Lucong Wang, Yiyin Zhang

**Affiliations:** ^1^Department of Hospital Infection Control, Tongde Hospital of Zhejiang Province, Hangzhou, China; ^2^Department of Hospital Infection Control, The First Affiliated Hospital of Zhejiang Chinese Medical University (Zhejiang Provincial Hospital of Traditional Chinese Medicine), Hangzhou, China; ^3^Department of Critical Care, The First Affiliated Hospital of Zhejiang Chinese Medical University (Zhejiang Provincial Hospital of Traditional Chinese Medicine), Hangzhou, China; ^4^Department of Hospital Infection Control, Hangzhou Red Cross Hospital, Hangzhou, China; ^5^Department of Hospital Infection Control, Jindong District Chisong Town Central Hospital, Jinhua, China; ^6^Department of Hospital Infection Control, Ningbo Traditional Chinese Medicine Hospital, Ningbo, China

**Keywords:** influencing factor emergency support frontline healthcare workers, COVID-19, closed-loop management, infection prevention and control, influencing factor

## Abstract

**Background:**

This study aimed to understand the hospital-acquired COVID-19 infection rate and infection prevention and control status of emergency support frontline healthcare workers (ESFHCWs) under closed-loop management, and to explore the related factors affecting hospital-acquired COVID-19 prevention and control status.

**Methods:**

The study site was a provincial-level tertiary hospital in the Xinjiang Uygur Autonomous Region specializing in treating COVID-19 patients. ESFHCWs were assigned from different hospitals in Zhejiang Province to provide emergency medical support in this specialized hospital. All ESFHCWs were managed using a closed loop. A self-designed questionnaire was used to estimate basic information, work experience, and the status of infection prevention and control (SIPC). A total of 269 ESFHCWs responded to the questionnaire. A generalized linear regression model was used to estimate the factors influencing SIPC.

**Results:**

There were six hospital-acquired COVID-19 cases, with an infection rate of 2.23%. The independent risk factors influencing COVID-19 prevention and control status were work seniority, anxiety disorder, and consumption of gastrointestinal, anti-inflammatory and anti-asthmatic, and hypnotic sedative drugs. Compared with ESFHCWs with more than 10 years of work seniority, ESFHCWs with less than 5 years of work seniority and 5–10 years of work seniority had lower COVID-19 SIPC scores. Among ESFHCWs with anxiety disorder, the SIPC score was significantly lower than that of ESFHCWs without anxiety disorder. The SIPC scores of ESFHCWs taking other medications (gastrointestinal, anti-inflammatory and anti-asthmatic, and hypnotic sedative drugs) were lower than those of ESFHCWs who did not.

**Conclusion:**

The closed-loop management method may be effective in reducing the infection rate of hospital-acquired COVID-19 among ESFHCWs. HCWs with less than 10 years of work seniority, anxiety disorder, and other medications (gastrointestinal, anti-inflammatory and anti-asthmatic, and hypnotic sedative drugs) were probably not suitable for participating in emergency assistant actions because of their poor SIPC scores. Further studies are needed to develop the selection criteria for ESFHCWs.

## 1. Introduction

Severe acute respiratory syndrome coronavirus 2 (SARS-CoV-2) infection (also known as COVID-19) was first reported in Wuhan city, China in 2019 and has spread to almost every country (1). Until February 10th in 2023, the total number of SARS-CoV-2 cases was 755,385,709, with a mortality rate of 6,833,388 (0.90%) (2).

Frontline healthcare workers (HCWs) are at higher risk of infection than the general population. Previous reports have shown that 1,716 medical staff members were infected with COVID-19 in Wuhan during the early stages of the outbreak (3). In Ethiopia, at least 1,311 HCWs were infected as of September 17, 2020 (3). From January to June 2022, 64.4% of the 284 HCWs were infected with COVID-19 in Kuwait (4). The main influencing factors of COVID-19 infection among HCWs are being unvaccinated (5), having colleagues or family members with COVID-19 (4), are intensive care unit (ICU) department workers, failure to wear masks (6) or personal protective equipment (PPE), unreasonable work schedules (7), lack of access to hand sanitizers, providing care within 1 m for COVID-19 patients, and direct contact to an environment in which a patient with COVID-19 received care (3).

Before December 2022, China maintained a strict COVID prevention and control policy, called the dynamic clearing policy (8), which involves stamping out an outbreak whenever it occurs and taking action in a relatively short timeframe to avoid community spread. This strategy did not eliminate all viruses but enabled detection of the epidemic in the early stages and isolation of potential cases. In November 2022, owing to the COVID-19 outbreak in the Xinjiang Uygur Autonomous Region, medical resources were in short supply. A medical team formed by multiple units was dispatched to assist a designated hospital in the region to assist in the treatment of patients infected with COVID-19.

There have been many reports (3–7) of HCWs infected with COVID-19. In previous studies, it was difficult to determine the source of infection, and it was impossible to exclude the influence of community transmission. In this study, all ESFHCWs were under closed-loop management and provided direct care to patients confirmed to have developed COVID-19. They may have a higher risk of contracting the SARS-CoV-2 infection (9) and require specific infection prevention capabilities and good physical and psychological status (10). This study aimed to understand the hospital-acquired COVID-19 infection rate and infection prevention and control status of ESFHCWs under closed-loop management, and to explore the related factors affecting hospital-acquired COVID-19 prevention and control status.

## 2. Methods

### 2.1. Sample

The study site was a provincial-level tertiary hospital in the Xinjiang Uygur Autonomous Region specializing in treating COVID-19 patients. The ESFHCWs who assisted this designated hospital were assigned from different hospitals in Zhejiang Province and specialized in providing emergency medical support. A medical team was selected as the study sample. Almost 300 HCWs from 63 different hospitals/institutions in Zhejiang Province were sent to the site within 3 days. Before this mission, they worked in different departments of health facilities, such as respiratory, infection, emergency, intensive care units (ICU), and hospital infection management departments. In responding to the sudden outbreak of COVID-19, China has worked to prevent both imported cases and domestic resurgences for almost 3 years. All HCWs in different departments have experienced repeated COVID-19 training and assessment and have relevant knowledge. All ESFHCWs participating in this emergency mission were trained and qualified in the *COVID-19 Prevention and Control-Consensus Diagnosis and treatment of COVID-19 (9th trial edition)* (11), including the selection of PPE, the wearing and removing of PPE, hand hygiene, isolation requirements, COVID-19 diagnosis and treatment, and other related content before coming to Xinjiang Uygur Autonomous Region. Intensive care professionals are required to have rich experience in treatment and nursing, and to be able to carry out treatment and nursing work independently. The selected personnel should have good will quality, a sense of responsibility, meet professional requirements, strong business ability, good health, and be able to undertake high-intensity medical treatment. Priority should be given to the experience of participating in the treatment of COVID-19. Unfortunately, considering the time urgency of this task, these selection criteria may not be well implemented, and these ESFHCWs may not fully meet the inclusion criteria.

### 2.2. Closed-loop management

The medical team was comprised a leader and a management team (medical group, nursing group, hospital infection management group, and logistics support group) and strictly implemented closed-loop management for ESFHCWs and hotel staff. Closed-loop management refers to point-to-point (hotel-hospital) docking, except for medical team members and hotel staff; i.e., there is no contact with other personnel or social activity tracking of a management approach. Each person lived in a single room. Medical, nursing, and hospital infection management groups are responsible for medical-related work, including routine medical services, body temperature monitoring, nucleic acid sampling, personnel transport, health education, and personal protection training in designated hospitals. The logistics support team is responsible for protecting lives and other materials. The hotel staff carries out the cleaning and disinfection of the internal and external environments (including the commuter bus), and the hospital infection group provides training guidance. The management team established a full-time accommodation manager to manage the 24 h access of residents.

ESFHCWs treated COVID-19 patients in five isolation wards of the designated hospital, including four general wards and one ICU. ESFHCWs implement reasonable scheduling and arrange rotation breaks. Each person enters the isolation ward once per day, and the working time is less than 4 h. These medical staff tested negative for SARS-CoV-2 nucleic acid for three consecutive days before entering the designated hospital and were provided with sufficient protective equipment. Additionally, they received unified training and assessment of infection prevention and control-related knowledge, such as wearing and removing PPE and occupational exposure disposal processes. Only after passing the evaluation, they could initiate working in the designated hospital. They were required to wear N95 respirators outside the hotel room and all other PPE (including protective coveralls, latex gloves, face shields, and shoe covers) before entering the hospital. Infection prevention and control training throughout working hours and daily supervision reminders were carried out through video surveillance during work. Throat swabs from ESFHCWs were collected daily for COVID-19. All HCWs were tested for COVID-19 every day.

### 2.3. Questionnaire

This study used an electronic questionnaire administered to investigate WeChat platforms. The questionnaire consisted of basic information, work experience, and the SIPC of ESFHCWs. Basic information included sex, age, marital status, SARS-CoV-2 vaccination, basic diseases, taking drugs, whether infected with SARS-CoV-2, and time of infection detection. Work experience includes the original medical institutions level, academic major, educational background, professional titles, position, work seniority, department, and whether the hospital has anti-epidemic experience. The SIPC of ESFHCWs includes data on infection prevention and control ability (training received, awareness of infection prevention and control, wearing and removal of PPE, supervision, and remindings) and physiological and psychological status since arrival to the Xinjiang Uygur Autonomous Region (personal emotion, psychological pressure, appetite, sleep, defecation, and medication). The SIPC part of questionnaire with 29 questions was designed based on semantic differential (SD) scaling (12). Five selection intervals were set for each question, which was 1–5, with a total score of 145. The higher the score, the better the SIPC of ESFHCWs.

### 2.4. Ethical considerations

Approval from the ethical review board of the First Affiliated Hospital of Zhejiang Chinese Medical University has been granted (2023-KSL-230-01). Before the study, the leaders of this medical team had given the consent and agreed cooperation. Before answering the questionnaire, all potential participants were informed verbally and given a chance to ask questions. In accordance with the general data protection regulation, the participants have been informed of their right to withdraw at any time and of how their data will be managed. All questionnaires were self-filled by participants anonymously. All records were identified by a code number. All local databases were password protected. To ensure confidentiality, data shared with project team members were blinded to any identifying participant information.

### 2.5. Data analysis

Statistical Package for the Social Sciences (SPSS) 24.0, a statistical software package, was used for testing the reliability and validity test of the questionnaire, general descriptive statistical analysis, and generalized linear regression analysis. Shapiro–Wilk and a histogram were used to test the normality of the data in this study. Frequency (N) and percentage (%) were used as categorical variables. Means and standard deviations were calculated for numerical variables. The Mann–Whitney *U* test and Kruskal–Wallis *H* test were used for comparison of differences in the scores of ESFHCWs with different characteristics in terms of SIPC. Variables were considered significant at a *p*-value less than 0.05.

## 3. Results

### 3.1. Reliability, validity

The Cronbach’s alpha for the questionnaire showed acceptable reliability (*α* = 0.778). Exploratory factor analysis was used for validity analysis with Kaiser–Meyer–Oklin (KMO) measuring 0.628 and Bartlett’s test of sphericity being significant (significant *p* < 0.001) and the cumulative variance interpretation rate was 74.641%, indicating that the research data had good structural validity.

### 3.2. Basic information

Basic information on the study participants is presented in Table 1. A total of 269 ESFHCWs who responded to the questionnaire were included. The mean age of the participants was 33.4 years, ranging from 22–57 years. Higher percentage of women (72.1%) participated in the study than men, in which 37.92% of the ESFHCWs were married and 68 (25.28%) were from medical institutions below grade three. The nursing profession (73.6%) accounted for the largest proportion, and 192 (71.38%) of the participants had a bachelor’s degree. There were 240 (89.21%) professional titles, mainly intermediate and below. Most staffs were doctors and nurses (202; 75.09%). Over 78.44% of ESFHCWs had a work seniority of more than 5 years. More than 60% of ESFHCWs had worked in an ICU and respiratory infections and hospital infection management departments. Approximately half of the ESFHCWs were experienced in anti-epidemic support, 254 (94.42%) were vaccinated with three or more doses of the SARS-CoV-2 vaccine, 37 (13.75%) had underlying diseases, and 179 (66.55%) had taken drugs during their work. There were six ESFHCWs (2.23%) with nosocomial SARS-CoV-2 infection.

**Table 1:** Baseline characteristics and difference in the SIPC scores of HCWs (*n* = 269).

Characteristics	*N*	Percentage (%)	Score	*Z*/*H*	*p*
Mean age (SD), min–max	33.40 (6.47), 22–57		125.39 ± 8.08		
*Sex*					
Male	75	27.88	123.97 ± 8.91	1.48	0.14
Female	194	72.12	125.93 ± 7.69		
*Marital status*					
Married	102	37.92	124.65 ± 8.00	1.12	0.26
Single	167	62.08	125.84 ± 8.12		
*SARS-CoV-2 vaccination (times)*					
0–2	15	5.58	125.53 ± 7.92	-0.00	1.00
3/>3	254	94.42	125.38 ± 8.11		
*Underlying diseases(yes/no)*					
Immune diseases or not	2/267	0.74/99.26	116.00 ± 2.83/125.46 ± 8.07	−1.83	0.07
Allergic diseases or not	15/254	5.58/94.42	124.33 ± 7.45/125.45 ± 8.13	−0.90	0.37
Diabetes or not	2/267	0.74/99.26	132.50 ± 2.12/125.33 ± 8.09	1.54	0.12
Digestive system diseases or not	5/264	1.86/98.14	118.00 ± 10.61/125.53 ± 7.99	−1.61	0.11
Endocrine diseases or not	4/265	1.49/98.51	117.75 ± 8.66/125.50 ± 8.04	−1.92	0.06
Anxiety disorder^a^ or not	1/268	0.37/99.63	98/125.49 ± 7.92	−1.73	0.01
Hypertension or not	5/264	1.86/98.14	131 ± 5.43/125.28 ± 8.09	1.68	0.09
Lumbar disc herniation or not	1/268	0.37/99.63	120/125.41 ± 8.09	−0.95	0.45
Asthma or not	2/267	0.74/99.26	118.50 ± 12.02/125.44 ± 8.06	−1.00	0.32
*Taking drugs(yes/no)*					
Cold medicine or not	37/232	13.75/86.25	123.32 ± 8.19/125.72 ± 8.03	1.68	0.09
Gastrointestinal drugs or not	17/252	6.32/93.68	119.00 ± 9.59/125.82 ± 7.81	2.90	0.00
Anti-diarrheal drugs or not	10/259	3.72/96.28	124.70 ± 9.08/125.41 ± 8.06	0.12	0.90
Painkillers or not	12/257	4.46/95.54	121.58 ± 9.76/125.56 ± 7.97	1.43	0.15
Anti-inflammatory/anti-asthmatic drugs or not	9/260	3.35/96.65	118.89 ± 7.11/125.61 ± 8.03	2.61	0.01
Hypnotic sedative drugs or not	57/212	21.19/78.81	122.30 ± 7.62/126.22 ± 8.02	3.53	0.00
Anti-allergic drugs or not	12/257	4.46/95.54	121.42 ± 8.23/125.57 ± 8.04	1.74	0.08
Antibiotics or not	10/259	3.72/96.28	124.40 ± 9.83/125.42 ± 8.03	0.16	0.87
Others^b^ or not	15/254	5.58/94.42	121.93 ± 8.23/125.59 ± 8.04	1.69	0.09
*Infected with SARS-CoV-2*					
Yes	6	2.23	127.17 ± 6.80	0.42	0.67
No	263	97.77	125.35 ± 8.12		
*Original medical institutions level*					
Tertiary hospital	201	74.72	125.60 ± 7.94	−0.62	0.53
Hospitals below grade three	68	25.28	124.75 ± 8.53		
*Academic major*					
Medicine	71	26.39	123.55 ± 8.62	2.20	0.03
Nursing	198	73.61	126.05 ± 7.80		
*Educational background*					
Junior college or below	30	11.15	125.00 ± 8.20	0.27	0.87
Undergraduate	192	71.38	125.42 ± 7.90		
Master’s degree or above	47	17.47	125.51 ± 8.88		
*Professional titles*					
Junior titles or below	123	45.72	124.73 ± 8.15	3.03	0.22
Intermediate titles	117	43.49	125.59 ± 8.12		
Senior titles	29	10.78	127.34 ± 7.50		
*Position*					
Ordinary doctors	45	16.73	122.76 ± 8.30	9.80	0.02
Ordinary nurses	157	58.36	125.51 ± 8.07		
Middle/senior cadres	57	21.19	126.19 ± 7.38		
Others^c^	10	3.72	130.70 ± 8.45^d^		
*Work seniority (in years)*					
<5	58	21.56	122.74 ± 8.91	11.40	0.00
5–10	82	30.48	124.78 ± 7.54		
>10	129	47.96	126.96 ± 7.71^e^		
*Work experience(department)*					
*ICU or not*					
Yes	103	38.29	124.89 ± 7.16	1.39	0.17
No	166	61.71	125.69 ± 8.61		
*Respiratory department or not*					
Yes	41	15.24	124.29 ± 7.88	1.10	0.27
No	228	84.76	125.58 ± 8.12		
*Infection department or not*					
Yes	14	5.20	123.71 ± 6.80	1.06	0.29
No	255	94.80	125.48 ± 8.15		
*Hospital infection management department or not*					
Yes	5	1.86	133 ± 5.00	−2.34	0.02
No	264	98.14	125.24 ± 8.07		
*Anti-epidemic experience* ^f^					
Yes	150	55.76	125.67 ± 8.38	−1.00	0.32
No	119	44.24	125.03 ± 7.72		

SARS-CoV-2, severe acute respiratory syndrome coronavirus 2; ICU, intensive care unit; SD, standard deviation.

a Anxiety disorder-as a underlying diseases, which was clearly diagnosed before coming to Xinjiang Uygur Autonomous Region.

b Others-adrenocorticosteroids, laxatives, hormone drugs, vitamin drugs, angina medicine, anti-depressants, and hypotensive drugs.

c Others-full-time staff of the hospital infection management department, logistics support personnel.

d Compared with ordinary doctors, *p* < 0.05.

e Compared with less than 5 years of work seniority, *p* < 0.05.

f Anti-epidemic experience-participated in COVID-19 nucleic acid sampling or COVID-19 treatment.

The average SIPC score of ESFHCWs was 125.39 ± 8.08. The data in this investigation did not conform to the normal distribution, according to the normality test. The results of Mann–Whitney *U* test and Kruskal–Wallis *H* test showed that there were statistically significant differences in the SIPC scores of ESFHCWs for anxiety disorder (*Z*: −1.73; *p* < 0.05), taking gastrointestinal (*Z*: 2.90; *p* < 0.05), anti-inflammatory, anti-asthmatic (*Z*: 2.61; *p* < 0.05) and hypnotic sedative (*Z*: 3.53; *p* < 0.05) drugs, academic major (*Z*: 2.20; *p* < 0.05), position (*H*: 9.80; *p* < 0.05), work seniority (*H*: 11.40; *p* < 0.05), and work experience in the hospital infection management department (*Z*: −2.34; *p* < 0.05). According to the results of the academic major, ESHCWs with anxiety disorder, and taking other medications (gastrointestinal, anti-inflammatory and anti-asthmatic, and hypnotic sedative drugs) had lower scores. The total score of infection prevention and control status of nurses (126.05 ± 7.80) was higher than that of doctors (123.55 ± 8.62). Compared with the full-time staff of the hospital infection management department and logistics support personnel (130.70 ± 8.45), the ESFHCWs with the position of ordinary doctors (122.76 ± 8.30) scored lower in the infection prevention and control status. Compared with less than 5 years of work seniority (122.74 ± 8.91), the ESFHCWs with more than 10 years of work seniority (126.96 ± 7.71) had higher scores. Being a ESFHCW with work experience in the hospital infection management department (133 ± 5.00) were found related to scoring significantly higher points from the questionnaire. Besides, the score of the six infected patients was 127.17 ± 6.80, no less than 125.35 ± 8.12 of the uninfected patients, and the difference was not statistically significant (*Z*: 0.42; *p* > 0.05).

Basic information on the six ESFHCWs with nosocomial SARS-CoV-2 infection, of which two were male doctors and four were nurses, is presented in Table 2. The mean age of the infected individuals was 29.43 years, ranging from 22–34 years. Three of them were from medical institutions below grade three. Four of the participants had undergraduate educational backgrounds. All the participants had intermediate and below-professional titles. Four had work experience in intensive care and respiratory departments, and two had no experience of anti-epidemic support. One patient with type 2 diabetes did not complete the booster dose vaccination. Half of the patients were taking hypnotic sedative drugs. The shortest infection time was only 1 day.

**Table 2:** Basic characteristics of six patients infected with SARS-CoV-2.

No.	1	2	3	4	5	6
Age (in years)	34	33	22	29	34	24
Sex	Male	Female	Male	Male	Male	Female
Marital status	Married	Married	Single	Single	Married	Single
Original medical institutions level	Hospitals below grade three	Tertiary hospital	Tertiary hospital	Tertiary hospital	Hospitals below grade three	Hospitals below grade three
Academic major	Medicine	Nursing	Nursing	Nursing	Medicine	Nursing
Educational background	Undergraduate	Undergraduate	Junior college or below	Undergraduate	Undergraduate	Junior college or below
Professional titles	Intermediate titles	Intermediate titles	Junior titles or below	Junior titles or below	Intermediate titles	Junior titles or below
Position	Ordinary doctors	Middle/senior cadres	Ordinary nurses	Ordinary nurses	Ordinary doctors	Ordinary nurses
Work seniority (in years)	>10	>10	<5	5–10	5–10	<5
Work experience	Respiratory department	ICU	ICU	ICU	—	—
Anti-epidemic experience	No	Yes	Yes	No	Yes	Yes
SARS-CoV-2 vaccination (times)	0–2	3/>3	3/>3	3/>3	3/>3	3/>3
Basic diseases	Diabetes	—	—	—	—	—
Taking drugs	Others (Adrenocorticosteroids)	Hypnotic sedative drugs/anti-allergic drugs	—	Hypnotic sedative drugs	Hypnotic sedative drugs	—
Time of infection detected (in days)	1	13	13	14	14	18

SARS-CoV-2, severe acute respiratory syndrome coronavirus.

### 3.3. Influencing factors associated with the SIPC scores of ESFHCWs

Generalized linear regression analysis was performed on the SIPC scores of ESFHCWs with different characteristics. The results are summarized in Table 3. Work seniority; anxiety disorder; and use of gastrointestinal, anti-inflammatory and anti-asthmatic, and hypnotic sedative drugs were the independent risk factors affecting the COVID-19 prevention and control scores of ESFHCWs. Compared with ESFHCWs with more than 10 years of work seniority, ESFHCWs with less than 5 years of work seniority [*β*: −0.040; 95% confidence interval (CI): −0.0596 to −0.0204] and 5–10 years of work seniority [*β*: −0.022; 95% confidence interval (CI): −0.0396 to −0.0044] had lower COVID-19 SIPC scores. These scores were higher in ESFHCWs without anxiety disorder [*β*: 0.201; 95% confidence interval (CI): 0.0775–0.3245] compared to those with anxiety disorder. And these scores were lower in ESFHCWs taking any gastrointestinal [*β*: −0.027; 95% confidence interval (CI): −0.0446 to −0.0094], anti-inflammatory, anti-asthmatic [*β*: −0.043; 95% confidence interval (CI): −0.0744 to −0.0116], and hypnotic sedative [*β*: −0.061; 95% confidence interval (CI): −0.1022 to −0.0198] drugs compared to those not consuming the abovementioned drugs.

**Table 3:** Generalized linear regression analysis of the SIPC scores among healthcare workers with different characteristics.

Variables		Coefficient	Standard error	*t*	*p*	95% CI
*Work seniority (in years)*						
<5	−0.040	0.010	−4.14	<0.001	−0.0596	−0.0204
5–10	−0.022	0.009	−2.51	0.013	−0.0396	−0.0044
>10	0					
*Anxiety disorder*						
No	0.201	0.063	3.17	0.002	0.0775	0.3245
Yes	0					
*Gastrointestinal drugs*						
Yes	−0.027	0.009	−2.99	0.003	−0.0446	−0.0094
No	0					
*Anti-inflammatory/anti-asthmatic drugs*						
Yes	−0.043	0.016	−2.74	0.007	−0.0744	−0.0116
No	0					
*Hypnotic sedative drugs*						
Yes	−0.061	0.021	−2.96	0.003	−0.1022	−0.0198
No	0					

CI, confidence interval.

## 4. Discussion

As the location of this study was a designated hospital for COVID-19, high exposure to SARS-CoV-2 was predictable. HCWs are reportedly infected after contacting COVID-19 patients (13, 14). Compared with workplace exposure, community risk factors were more strongly correlated with the positive rate of SARS-CoV-2 in HCWs (15). All relevant personnel (including ESFHCWs, logistics support personnel, and cleaning personnel) assigned to the Xinjiang Uygur Autonomous Region underwent closed-loop management throughout the entire process; i.e., there was no contact with outside personnel, and they traveled between the rest station and the hospital via a two-point one-line dedicated commuter bus. Therefore, the possibility of a community infection can be ruled out.

There were six SARS-CoV-2 hospital infection cases during the study of the designated hospital, and the infection rate was 2.23%. Reportedly, 17.14% (18/105) of medical staff were serologically positive after contacting patients diagnosed with novel coronavirus (14), our infection control measures may be effective in reducing the infection rate. Through closed-loop management, reasonable working hours, continuous infection prevention and control training, and an adequate supply of PPE (16), the risk of ESFHCWs’ exposure to SARS-CoV-2 could be effectively reduced. One doctor was infected only 1 day after work, which may be related to the fact that he had type II diabetes and did not receive the third dose of a COVID-19 vaccine (17–20).

Also, in our study, we found that the total score of infection prevention and control status of nurses was higher than that of doctors, and the difference was statistically significant, suggesting that the infection prevention and control status of doctors should be improved. On the one hand, this phenomenon may be related to the small number of doctors involved in the survey. On the other hand, it may be related to the strict compliance with the aseptic operation standard process and the strict implementation of the preventive health care system emphasized by nurses in the daily operation of nursing (21). At the same time, it can be expected that ESFHCWs with work experience in hospital infection management departments generally have higher scores, and the difference was statistically significant. Additionally, the scores of infected people are higher than those of uninfected people, and the difference was not statistically significant, indicating that accidental occupational exposure may be inevitable (22). This challenge has yet to be addressed.

Generalized linear regression analysis of the SARS-CoV-2 infection prevention and control status of ESFHCWs with different characteristics revealed that work seniority; anxiety disorder; and use of gastrointestinal, anti-inflammatory, anti-asthmatic, and hypnotic sedative drugs were the independent risk factors affecting the COVID-19 prevention and control status of ESFHCWs. Considering our findings, one can see that ESFHCWs with 5 years or less of work seniority have lower score of infection prevention and control status. Correspondingly, the ESFHCWs who have worked for more than 10 years have the highest score of infection prevention and control status, and the best infection prevention and control status. ESFHCWs with short work seniority may have just entered the workplace from the learning environment of medical colleges and hence lack practical experience in infection prevention and control, particularly in a pandemic situation. Therefore, they are more prone to fear, stress, insomnia (23), and depression (24), which may affect their performance at the workplace. ESFHCWs with anxiety disorder may experience cognitive decline (25), which may affect their knowledge and ability to prevent and control infections. They are more likely to make mistakes in clinical operations and infection prevention and control, and the risk of occupational exposure increases owing to constipation, insomnia, and transmission. Consuming gastrointestinal, anti-inflammatory, anti-asthmatic, and hypnotic sedative drugs reflects a lower general health of ESFHCWs and sleep-associated problems (26). ESFHCWs who use gastrointestinal drugs may become unaccustomed to the local diet, resulting in gastrointestinal dysfunction. SARS-CoV-2 is primarily transmitted through the respiratory tract (27). ESFHCWs using anti-inflammatory and anti-asthmatic drugs may have respiratory diseases or symptoms that greatly increase the risk of infection prevention and control. ESFHCWs have poor sleep quality or insomnia in high-pressure working environments (28), which is reflected by the use of hypnotic sedative drugs.

It is recommended that screening criteria for team members be developed in advance when establishing emergency medical teams to reduce the impact of these factors. HCWs with short work experience, anxiety disorder, and a tendency to use gastrointestinal, anti-inflammatory, anti-asthmatic, and hypnotic or sedative drugs may not be suitable for participation in COVID-19 medical assistance. Moreover, we suggest that after the formation of the team, the team members with these factors should be further evaluated according to the work arrangements and assignments. Based on the work experience of team members, HCWs with higher work experience should guide and carry out the medical treatment by leading in groups. Simultaneously, the training and supervision of team members regarding the aforementioned factors should be strengthened to improve infection prevention and control. Work shifts should be adjusted and rest or transfer from the current position should be arranged in a timely manner when physical or psychological symptoms are experienced by HCWs. Mental and material support should be provided to medical staff during emergencies to meet the needs of HCWs.

## 5. Conclusion

The closed-loop management method may be effective in reducing the infection rate of hospital-acquired COVID-19 among ESFHCWs. HCWs with less than 10 years of work seniority, anxiety disorder, and other medications (gastrointestinal, anti-inflammatory and anti-asthmatic, and hypnotic sedative drugs) were probably not suitable for participating in emergency assistant actions because of their poor SIPC scores. Further studies are needed to develop the selection criteria for ESFHCWs.

## Data availability statement

The raw data supporting the conclusions of this article will be made available by the authors, without undue reservation.

## Ethics statement

The studies involving human participants were reviewed and approved by the ethical review board of the First Affiliated Hospital of Zhejiang Chinese Medical University, which was affiliated to the First Affiliated Hospital of Zhejiang University of Traditional Chinese Medicine. Written informed consent from the participants was not required to participate in this study in accordance with the national legislation and the institutional requirements.

## Author contributions

ML, GX, and TG contributed to the conception and design of the study. GX organized the database. ML performed the statistical analysis and wrote the first draft of the manuscript. ZZ, JW, TH, ML, LW, and YZ wrote sections of the manuscript. All authors contributed to the article and approved the submitted version.

## Conflict of interest

The authors declare that the research was conducted in the absence of any commercial or financial relationships that could be construed as a potential conflict of interest.

## Publisher’s note

All claims expressed in this article are solely those of the authors and do not necessarily represent those of their affiliated organizations, or those of the publisher, the editors and the reviewers. Any product that may be evaluated in this article, or claim that may be made by its manufacturer, is not guaranteed or endorsed by the publisher.
